# Feasibility of electronic nose technology for discriminating between head and neck, bladder, and colon carcinomas

**DOI:** 10.1007/s00405-016-4320-y

**Published:** 2016-10-11

**Authors:** R. M. G. E. van de Goor, N. Leunis, M. R. A. van Hooren, E. Francisca, A. Masclee, B. Kremer, K. W. Kross

**Affiliations:** 1 Department of Otorhinolaryngology, Head and Neck Surgery, Maastricht University Medical Center, PO Box 5800, 6202 AZ Maastricht, The Netherlands; 2 Department of Urology, Maastricht University Medical Center, Maastricht, The Netherlands; 3 Department of Gastroenterology and Hepatology, Maastricht University Medical Center, Maastricht, The Netherlands

**Keywords:** Electronic nose technology, Head and neck cancer, Colon cancer, Bladder cancer, Volatile organic compounds, Diagnosis

## Abstract

Electronic nose (e-nose) technology has the potential to detect cancer at an early stage and can differentiate between cancer origins. Our objective was to compare patients who had head and neck squamous cell carcinoma (HNSCC) with patients who had colon or bladder cancer to determine the distinctive diagnostic characteristics of the e-nose. *Feasibility study* An e-nose device was used to collect samples of exhaled breath from patients who had HNSCC and those who had bladder or colon cancer, after which the samples were analyzed and compared. One hundred patients with HNSCC, 40 patients with bladder cancer, and 28 patients with colon cancer exhaled through an e-nose for 5 min. An artificial neural network was used for the analysis, and double cross-validation to validate the model. In differentiating HNSCC from colon cancer, a diagnostic accuracy of 81 % was found. When comparing HNSCC with bladder cancer, the diagnostic accuracy was 84 %. A diagnostic accuracy of 84 % was found between bladder cancer and colon cancer. The e-nose technique using double cross-validation is able to discriminate between HNSCC and colon cancer and between HNSCC and bladder cancer. Furthermore, the e-nose technique can distinguish colon cancer from bladder cancer.

## Introduction

The leading cause of death worldwide is cancer, with about 14.1 million new cases and 8.2 million deaths in 2012 [1]. The number of new cases is expected to rise to 22 million within the next two decades [2]. Only early detection and treatment can reduce the mortality rate [3]. That requires a quick, reliable, non-invasive, and inexpensive way to screen for cancer so that treatment might start at the earliest possible stage of the disease. Early diagnosis could lead to better radical treatment, less loss of function, and a higher survival rate.

Exhaled human breath contains hundreds of volatile organic compounds (VOCs) that can be detected by gas chromatography and mass spectrometry (GC–MS) on the compound level and by pattern recognition with electronic nose (e-nose) technology. There are three types of exhaled VOCs. Local VOCs arise directly in the alveoli or the airway lumen along the respiratory tract. Exogenous VOCs are ‘inhaled’ or absorbed through the skin. Some VOCs, originating from metabolic processes in the body, dissolve in the blood, subsequently exit the circulation and enter the respiratory tract through the alveoli [4].

Applications of e-nose technology are common in the food and beverage industry, in monitoring air quality, and in the detection of explosive and chemical agents [5]. The interaction of VOCs with an array of partial selective chemical sensors (equivalent to the olfactory receptors in the human nose) results in a change in the resistance or conductance of the sensors. That change is transmitted to a processor. E-nose technology also has many health-care applications; among others, it is used for diagnosing colon cancer, chronic obstructive pulmonary disease (COPD), asthma, lung cancer, and head and neck cancer [6–10].

Recently, van Hooren et al. 2016 reported that the e-nose is able to discriminate between head and neck squamous cell carcinoma (HNSCC) and lung cancer [11]. That study used a handheld device with metal oxide sensors that are periodically heated when processing the breath sample. Oxidation or reduction of the VOCs present in the breath sample is measured while the resistance changes as a function of temperature and time. Van Hooren et al. showed that e-nose technology, which uses VOC pattern recognition, was able to differentiate between HNSCC and lung cancer. Both types occur in the respiratory tract and share common risk factors such as smoking and male gender [12].

To our knowledge, no studies have been published on the use of e-nose technology to differentiate between HNSCC and bladder or colon cancer by means of VOC pattern recognition. Crucially, if a tool is used to screen for primary malignancies, it should be able to differentiate between tumors of different types in different compartments of the human body. Moreover, no other studies on this topic have described the double cross-validation model. That is a strategy to optimize the complexity of regression models and make a realistic estimate of prediction errors when the model is applied to new cases.

Against that backdrop, the main objective of the present study is to determine whether the e-nose technique is able to discriminate between HNSCC and bladder or colon cancer using double cross-validation. The secondary objective is to investigate whether the e-nose is able to discriminate between colon and bladder cancer. As such, the e-nose has potential in health care as a screening tool for different origins of cancer.

## Materials and methods

### Patients

This study was conducted in the Netherlands at a tertiary care referral hospital (Maastricht University Medical Center). It included patients with primary HNSCC originating from the oral cavity, pharynx, larynx, hypopharynx, nasopharynx, or nasal cavity. Also included were patients with primary cancer of the bladder or colon. The study protocol was approved by the medical ethics committee and was carried out in accordance with the Declaration of Helsinki.

The exclusion criteria were age under 18 years, tracheostomy, any treatment for current tumor, and a history of cancer. Patients initially enrolled were subsequently excluded when they did not or could not complete the 5-min measurement session or were unable to endure a nose clip. Malignancies of the salivary glands were also grounds for exclusion. The participants’ smoking habits and metabolic fasting state were documented. The latter was defined as no food or drink 4 h before the session, except for two units of non-caloric clear liquid 2 h prior to measurement. Smoking was defined as smoking in the previous month. Tumor characteristics and medical history were collected from the clinical records. For tumor staging, WHO classifications were used. Carcinomas in situ and non-invasive papillary bladder carcinomas were noted as stage 0 tumors. Any side or adverse effects during or shortly after measurement were documented. Informed consent was obtained from all patients.

### Study design

To acquaint the patients with the device, they received instructions for a test run of inhalations and exhalations. After the instructions, all patients were asked to inhale and exhale through the e-nose for 5 min. A clip was placed on the nose to prevent the entry of non-filtered air. Patients were instructed to enclose the lips by the mouthpiece at all times.

E-nose readings were synchronous with the regular diagnostic workup. Participants were not given any diagnostic information derived from their individual e-nose results. The routine diagnostic workup was based on national cancer guidelines and was independent of e-nose measurements. The e-nose outcomes were compared with histopathology from biopsies.

### Materials

The device used in this study (Aeonose; the eNose Company, Zutphen, the Netherlands) consists of three different micro-hotplate metal oxide sensors (AS-MLV sensors; Applied Sensors GmbH.). During the measurement, the hotplates are periodically heated and cooled between 260 and 340 °C in 32 steps during which the sensors are exposed to the exhaled breath. The reduction and oxidation (redox) reactions of the VOCs on the metal oxide surface affect the conductivity of the sensors. Over time, these changes create a unique pattern of redox reactions. (See van Hooren et al. 2016 for more details on the method) [11]. The measurements were performed with five Aeonoses (serial numbers 259, 309, 315, 362, and 379) to exclude possible machine-bound confounding factors.

### Statistical analysis

Differences in baseline characteristics were determined with the independent sample *t* test, Fisher’s exact test, or Pearson’s Chi square test. All statistical analyses were performed using IBM SPSS Statistics for Windows, Version 22.0 (IBM Corp.; Armonk, NY, USA).

Each e-nose measurement generates 64 (temperature values) times 36 (measurement cycles) times 3 (sensor) data points, forming a multi-way dataset consisting of conductivity values. After preprocessing, the data are compressed using a TUCKER3 solution for tensor decomposition. The vectors representing the coded patient information are subsequently used to train an artificial neural network (ANN). This training is carried out for a number of data scaling options, yielding different models for separating ‘HNSCC’ from ‘colon or bladder cancer’ patients. Data compression and ANN have been integrated in a proprietary software package (Aethena) of the eNose Company (Zutphen, the Netherlands). The binary results are presented in a scatter plot and a receiver operating characteristic curve (ROC curve). Matthews correlation coefficients (MCC) were calculated to determine the quality of the binary classifications and 95 % confidence intervals (CI) were given.

The data were labeled with the diagnosis of HNSCC, or colon cancer, or bladder cancer when processed in Aethena. The optimal results were obtained by combining multiple ANNs in the following sequence. First, one ANN separated all data into a positive and a negative group. Then each group was judged by three different ANNs, generating an average value of the ANN classifications (judge model). To calculate sensitivity, specificity, and overall accuracy for future, yet undefined breath samples, double cross-validation was performed. Using brute (computing) force, the optimal combination of available ANNs was determined. Double cross-validation ensures that comparable results can be expected when submitting blind data into the trained ANN.

## Results

One hundred and sixty-eight patients were included in this study. They had histopathologically proven HNSCC (*N* = 100), bladder cancer (*N* = 40), or colon cancer (*N* = 28). The tumor sites of the HNSCC patients were the oral cavity (*N* = 28), oropharynx (*N* = 23), nasopharynx/nasal cavity (*N* = 4), hypopharynx (*N* = 11), and larynx (*N* = 34). All HNSCC patients were diagnosed with squamous cell carcinoma (including three patients with squamous cell carcinoma in situ). In Table 1, the TNM stadiums of all HNSSC patients are shown.

**Table 1:** TNM staging of HNSCC patients

	0	CIS	1	2	3	4
T	/	3	28	30	19	20
N	60	/	13	25	2	/
M	95	/	5	/	/	/

### HNSCC and colon cancer

The baseline characteristics of the HNSCC vs. colon cancer patients are listed in Table 2. There are several baseline differences between the two groups: age (*p* = 0.024), currently smoking (*p* = 0.000), pack-years (*p* = 0.002), and tumor stage (*p* = 0.018). Of the 28 patients with colon cancer, 27 had an adenocarcinoma and one had a neuroendocrine carcinoma.

**Table 2:** Baseline characteristics of HNSCC and colon cancer

	HNSCC	Colon	*p* value	Test
Number of patients	100	28		
Age (years)	64	69	0.024′	†
Sex (male)	74	18	0.209	‡
Food intake <4 h (“Yes”)	23	4	0.162	‡
Currently smoking	57	4	0.000′	‡
Pack-years	34	17	0.002′	†
Aeonose serial number			0.276	*
259	27	6		
309	18	2		
315	18	10		
362	18	5		
379	19	5		
Tumor stage			0.018′	*
0	3	0		
1	26	6		
2	20	8		
3	16	11		
4	35	3		

* Pearson Chi square

^‡^Fisher’s exact test

^†^Independent *t* test

′ Significant

### HNSCC and bladder cancer

The baseline characteristics of the head and neck vs. bladder cancer patients are listed in Table 3. Several baseline differences were found: age (*p* = 0.020), food intake (*p* = 0.038), smoking (*p* = 0.002), and tumor stage (*p* = 0.000). There were 24 patients with a stage 0 tumor. Four patients had a carcinoma in situ, respectively, three with HNSCC and one bladder cancer patient. The remaining 20 patients had non-invasive papillary bladder carcinomas.

**Table 3:** Baseline characteristics of HNSCC and bladder cancer

	HNSCC	Bladder	*p* value	Test
Number of patients	100	40		
Age (years)	64	68	0.020′	†
Sex (male)	74	28	0.555	‡
Food intake <4 h (“Yes”)	64	23	0.038′	‡
Currently smoking	57	10	0.002′	‡
Pack-years	37	27	0.076	†
Aeonose serial number			0.223	*
259	27	5		
309	18	5		
315	18	8		
362	18	13		
379	19	9		
Tumor stage			0.000′	*
0	3	21		
1	26	6		
2	20	9		
3	16	2		
4	35	2		

* Pearson Chi square

^‡^Fisher’s exact test

^†^Independent *t* test

′ Significant

### Bladder cancer and colon cancer

Only one significant difference in baseline characteristic was found: tumor stage (*p* = 0.000). The data are presented in Table 4.

**Table 4:** Baseline characteristics of colon cancer and bladder cancer

	Colon	Bladder	*p* value	Test
Number of patients	28	40		
Age (years)	68	69	0.535	†
Sex (male)	18	30	0.246	‡
Food intake <4 h (“Yes”)	23	24	0.065	‡
Currently smoking	4	11	0.160	‡
Pack-years	40	28	0.106	†
Aeonose serial number			0.365	*
259	6	5		
309	2	5		
315	10	8		
362	5	13		
379	5	9		
Tumor stage			0.000′	*
0	0	21		
1	6	6		
2	8	9		
3	11	2		
4	3	2		

* Pearson Chi square

^‡^Fisher’s exact test

^†^Independent *t* test

′ Significant

### Data analysis

#### HNSCC and colon cancer

**Fig. 1:**
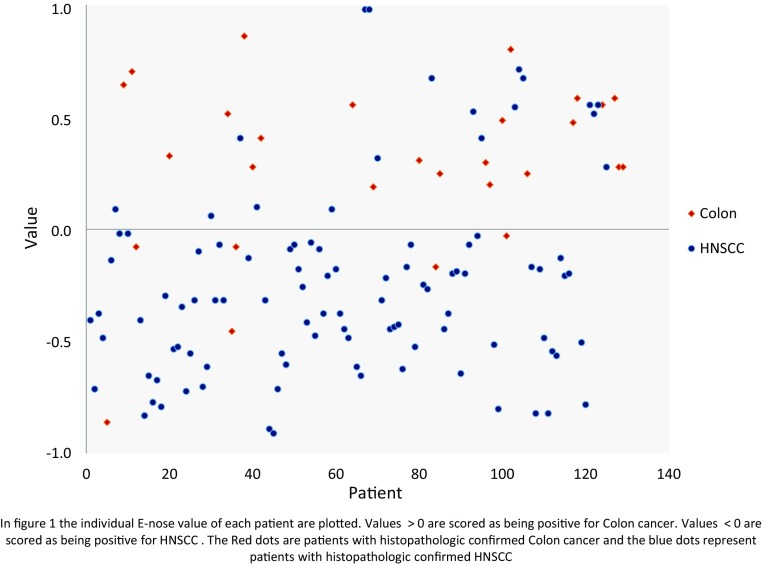
Scatter plot of HNSCC and colon cancer

**Fig. 2:**
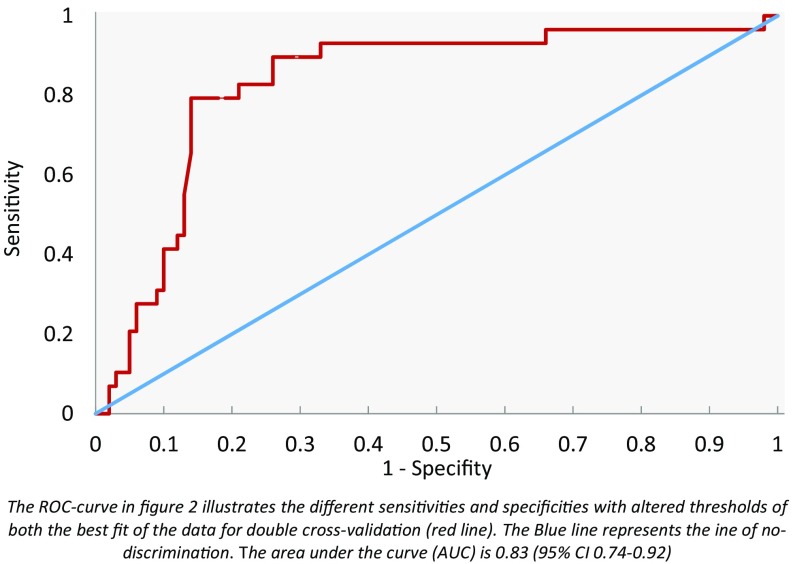
ROC curve of HNSCC and colon cancer

Figure 1 is a scatter plot of individual predictive values with a best fit of the data analyzed by the ANN. To obtain the best possible diagnostic accuracy of the data, the threshold was set to 0.00. This resulted in a sensitivity of 79 % and specificity of 81 %, with an overall accuracy of 81 % (MCC: 0.56) in differentiating between colon cancer and HNSCC. Cross-validation data are shown in Fig. 2.

#### HNSCC and bladder cancer

**Fig. 3:**
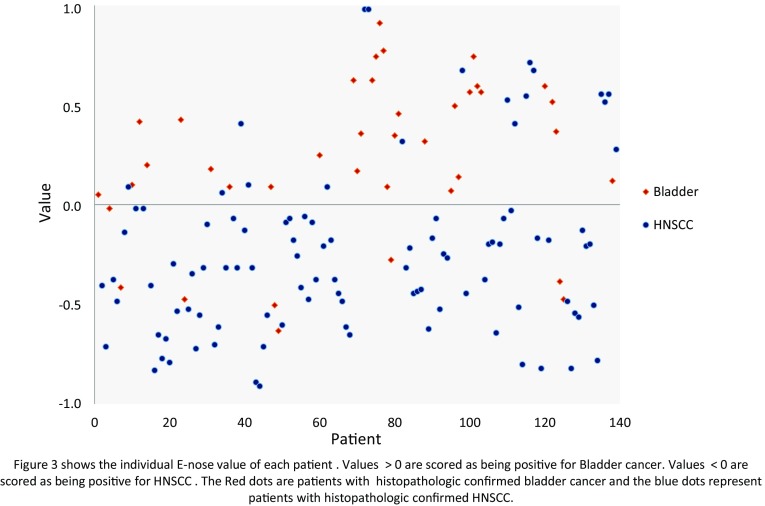
Scatter plot of HNSCC and bladder cancer

**Fig. 4:**
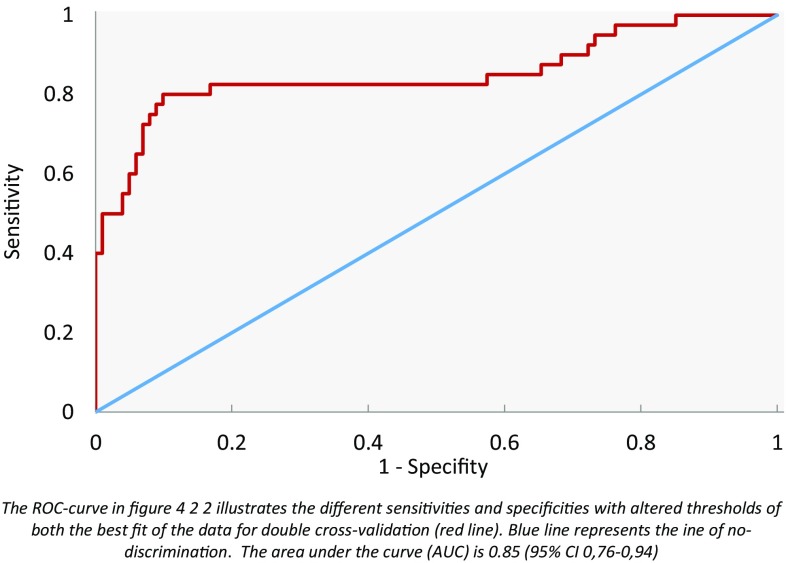
ROC curve of HNSCC and bladder cancer

Figure 3 is a scatter plot of individual predictive values with a best fit of the data analyzed by the ANN. To obtain the best possible diagnostic accuracy, the threshold was set to 0.00. The sensitivity was 80 % and specificity was 86 %, at an overall accuracy of 84 % (MCC: 0.66) in differentiating between colon carcinoma and HNSCC. Cross-validation data are given in Fig. 4.

#### Bladder cancer and colon cancer

**Fig. 5:**
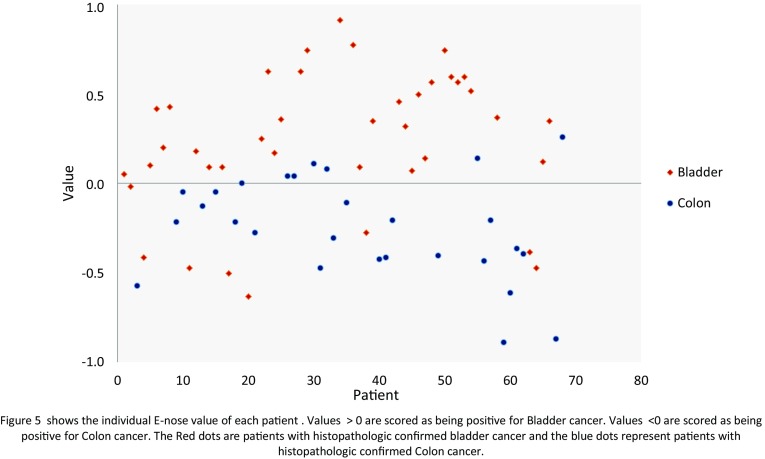
Scatter plot of bladder cancer and colon cancer

**Fig. 6:**
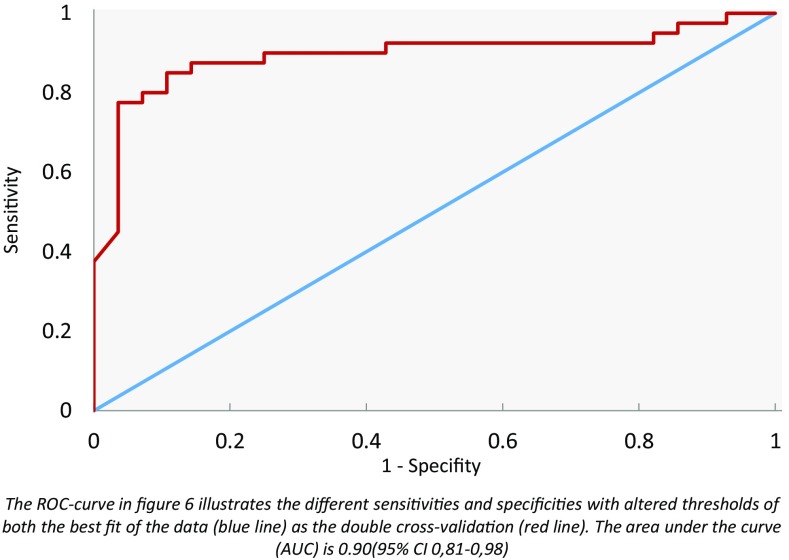
ROC curve of bladder and colon cancer

The scatter plot in Fig. 5 displays the individual predictive values with a best fit of the data analyzed by the ANN. For the best possible diagnostic accuracy, the threshold was set to 0.00. This resulted in a sensitivity of 88 % and specificity of 79 %, and an overall accuracy of 84 % (MCC: 0.69) in differentiating between colon carcinoma and bladder carcinoma. Cross-validation data are shown in Fig. 6.

## Discussion

In this feasibility study, the breath VOC patterns of patients with HNSCC were compared to the patterns of patients with colon or bladder cancer. Our results show that breath VOC pattern analysis with the e-nose is feasible. The technique exhibits a reasonable degree of sensitivity and specificity for double cross-validation when comparing HNSCC with colon cancer or bladder cancer.

Interest in the use of VOCs in diagnosing primary carcinomas has been growing. Meij et al. [6] tested 157 stool samples (40 patients with colon cancer, 60 patients with advanced adenomas, and 57 healthy controls). They found that the VOC profiles of patients with colon cancer differed significantly from those of controls without cancer (AUC 0.92, sensitivity 85 %, and specificity 87 %). Amel et al. [13] evaluated the breath VOC pattern analysis by testing 65 patients with colon cancer and 122 healthy controls. Their sensor analysis distinguished colon cancer from the healthy control group with 85 % sensitivity, 94 % specificity, and 91 % accuracy. Comparing HNSCC patients with healthy subjects, Gruber et al. [14] used an e-nose to analyze the breath samples of 22 patients with malignant larynx or pharynx tumors and 21 healthy controls. They were able to distinguish HNSCC patients from healthy controls as well as from individuals with benign tumors at a sensitivity of 77 %, specificity of 90 %, and overall accuracy of 83 %. Our group used an e-nose to evaluate VOC patterns in the exhaled breath of 36 HNSCC patients and 23 patients without malignant disease and found 90 % sensitivity and 80 % specificity in diagnosing HNSCC [10]. Using pattern recognition and principal component analysis (PCA), Peng et al. [15] showed that an e-nose can distinguish different tumors in different tracts (lung, colon, breast, prostate). Against that background, the innovative aspect of the present study is that double cross-validation is shown to improve the diagnostic accuracy when the generated Judge model is applied to new cases. Furthermore, this generated model can be translated to different Aeonose devices.

Double cross-validation showed a sensitivity of 79 % and specificity of 81 % when HNSCC was compared with colon cancer using breath samples and the e-nose. When comparing HSCNN with bladder cancer, this study found a sensitivity of 80 % and specificity of 86 %, and it showed a sensitivity of 88 % and specificity of 79 % when comparing bladder cancer with colon cancer.

In the past decade, diagnosis of primary cancers with VOCs has shown promising results. Among the various methods to analyze VOCs, one uses GC–MS and is able to identify specific volatile organic compounds for diseases of interest. However, this method has some disadvantages: cost, its time-consuming procedure, and the need for well-trained personnel to collect and analyze the samples. Furthermore, the identification of detected compounds is not straightforward; reference libraries have to be checked and validated using the mass and retention time of known standards.

Another method, the one used in this study, is e-nose technology, which is based on pattern recognition. The e-nose needs to be ‘trained’ to build a database for recognition, after which it can be used to classify blind samples. The crucial factors of meaningful pattern recognition are the size of the training set and representativeness of the sample for the populations to be tested. The advantage of the e-nose used in this study (Aeonose) is that it is a portable handheld device, making it easily applicable in an outpatient setting. Furthermore, the method is quick and fairly cheap.

### Limitations

The design of this feasibility study entailed some limitations; therefore, some caution should be taken when interpreting our results. First, there were significant baseline differences in both the HNSCC vs. colon cancer analysis and the HNSCC vs. bladder cancer analysis. These differences reflect the clinical setting: the majority of patients with HNSCC are smokers with an advanced tumor stage at first presentation [16]. Second, none of the patients with bladder or colon carcinoma had received a panendoscopy or any other diagnostic procedure to exclude HNSCC, as no clinical symptoms were present at the time of sample collection.

## Conclusion

The e-nose technique, using double cross-validation, is able to discriminate between HNSCC and colon cancer (sensitivity 79 %, specificity 81 %) and between HNSCC and bladder cancer (sensitivity 80 %, specificity 86 %). Furthermore, the e-nose can distinguish colon cancer from bladder cancer (sensitivity 88 %, specificity 79 %). Large, preferably blinded studies should be conducted to determine the role that the e-nose could play as a diagnostic tool in primary cancer diagnostics and management.
